# Agreement in cerebrovascular reactivity assessed with diffuse correlation spectroscopy across experimental paradigms improves with short separation regression

**DOI:** 10.1117/1.NPh.10.2.025002

**Published:** 2023-04-07

**Authors:** Kyle R. Cowdrick, Tara Urner, Eashani Sathialingam, Zhou Fang, Ayesha Quadri, Katherine Turrentine, Seung Yup Lee, Erin M. Buckley

**Affiliations:** a Georgia Institute of Technology and Emory University, Wallace H. Coulter Department of Biomedical Engineering, Atlanta, Georgia, United States; b Children’s Healthcare of Atlanta and Emory University School of Medicine, Department of Pediatrics, Atlanta, Georgia, United States; c Morehouse School of Medicine, Atlanta, Georgia, United States; d Kennesaw State University, Department of Electrical and Computer Engineering, Marietta, Georgia, United States; e Children’s Healthcare of Atlanta, Children’s Research Scholar, Atlanta, Georgia, United States

**Keywords:** cerebrovascular reactivity, diffuse correlation spectroscopy, cerebral blood flow

## Abstract

**Significance:**

Cerebrovascular reactivity (CVR), i.e., the ability of cerebral vasculature to dilate or constrict in response to vasoactive stimuli, is a biomarker of vascular health. Exogenous administration of inhaled carbon dioxide, i.e., hypercapnia (HC), remains the “gold-standard” intervention to assess CVR. More tolerable paradigms that enable CVR quantification when HC is difficult/contraindicated have been proposed. However, because these paradigms feature mechanistic differences in action, an assessment of agreement of these more tolerable paradigms to HC is needed.

**Aim:**

We aim to determine the agreement of CVR assessed during HC, breath-hold (BH), and resting state (RS) paradigms.

**Approach:**

Healthy adults were subject to HC, BH, and RS paradigms. End tidal carbon dioxide (EtCO_2_) and cerebral blood flow (CBF, assessed with diffuse correlation spectroscopy) were monitored continuously. CVR (%/mmHg) was quantified via linear regression of CBF versus EtCO_2_ or via a general linear model (GLM) that was used to minimize the influence of systemic and extracerebral signal contributions.

**Results:**

Strong agreement (CCC≥0.69; R≥0.76) among CVR paradigms was demonstrated when utilizing a GLM to regress out systemic/extracerebral signal contributions. Linear regression alone showed poor agreement across paradigms (CCC≤0.35; R≤0.45).

**Conclusions:**

More tolerable experimental paradigms coupled with regression of systemic/extracerebral signal contributions may offer a viable alternative to HC for assessing CVR.

## 1 Introduction

Cerebrovascular reactivity (CVR), defined as the ability of cerebral vasculature to dilate or constrict in response to a vasoactive stimulus, is an integral mechanism in brain homeostasis. Interest in CVR as a diagnostic and prognostic biomarker has accelerated in recent years because impaired CVR has been observed in numerous disease states, including cerebrovascular disease,^1^^–^^7^ stroke,^8^^–^^13^ cardiac arrest,^14^ and traumatic brain injury.^15^^–^^23^ Quantification of CVR coupled with interventions aimed at restoring abnormal CVR has the potential to reduce morbidity and mortality.

Several well-established experimental paradigms exist to study CVR non-invasively in humans. These methods typically involve quantification of the cerebral blood flow (CBF) response to changes in arterial carbon dioxide content via acetazolamide infusion, inhalation of carbon dioxide (CO_2_), or breath-holding. Intravenous acetazolamide administration has historically served as the preferred method to quantify CVR in clinical settings.^24^ Although the single dose injection is relatively simple, differences in pharmacokinetic/dynamic profiles among subjects lead to poor repeatability, and it is associated with a high incidence of side effects that preclude its modern use in many patient cohorts (e.g., children).^6^^,^^7^^,^^10^^,^^13^^,^^24^^,^^25^ In research settings, the most common method to quantify CVR is inhalation of CO_2_ to induce hypercapnia (HC), which is considered by many to be the “gold-standard” experimental paradigm. A standard HC challenge involves a limited duration inhalation of medical-grade ${\mathrm{CO}}_{2}$ (e.g., 5% ${\mathrm{CO}}_{2}$, 21% oxygen, and balance nitrogen) coupled with monitoring of CBF and end tidal CO_2_ (EtCO_2_). This approach is increasingly used due to the potency of CO_2_ as a vasodilator, rapid onset and cessation of the CBF response, and repeatability.^24^^,^^26^^–^^35^ However, HC requires complex gas delivery equipment, time, and expertise to perform the procedure, and it can induce anxiety in some subjects. Moreover, HC may be clinically contraindicated in patients with acute illness and/or compromised hemodynamics for whom the benefits of CVR assessment do not outweigh the risks of rapid or prolonged hyperemia (e.g., sickle cell disease).^36^ A breath-hold (BH) challenge, wherein a subject briefly holds their breath, offers a simpler alternative for manipulating arterial CO_2_. This approach offers many advantages over CO_2_ inhalation, including a lack of complex gas delivery equipment and improved patient tolerability given the self-directed nature of the BH.^37^^–^^40^ However, the BH approach requires an individual’s cooperation, which limits applicability in children <∼6y and in patients with severe conditions and entirely excludes its use in other cohorts (e.g., patients on a mechanical ventilator).

To bypass the limitations of existing experimental paradigms, recent investigations have explored the potential for measuring CVR using the intrinsic vasoactive stimulus of natural arterial CO_2_ fluctuations that occur when a subject is freely breathing.^29^^,^^40^^–^^44^ This resting state (RS) approach is attractive because it does not require patient compliance or intentional manipulation of arterial CO_2_. Thus, it has potential in those who have lost consciousness or who are sedated. However, the approach is thought to be susceptible to poor signal-to-noise due to the relatively small fluctuations in EtCO_2_ that occur during spontaneous breathing. Thus, CVR estimations at rest may be unreliable.^45^

Although the use of more tolerable experimental paradigms to assess CVR is clinically appealing, there has been limited investigation into the agreement of CVR measured by these approaches. Liu et al. recently demonstrated poor agreement of HC versus BH and of HC versus RS.^45^ The other handful of studies that have compared CVR across HC/BH/RS paradigms are limited in interpretation. These studies either compare relative changes in blood flow without accounting for differences in elicited EtCO_2_ response,^35^^,^^45^^,^^46^ which can vary appreciably across subjects and/or paradigms, or they focus on the spatial correlation of cohort-averaged, qualitative, regional CVR maps attained from two experimental paradigms.^40^^,^^47^^,^^48^ Given that these paradigms act on the vasculature in different ways and that each has relative merit in terms of robustness of response and ease of implementation, a systematic and rigorous comparison of CVR across paradigms is warranted.

Herein we quantify the agreement of CVR across HC, BH, and RS paradigms in healthy adults. To quantify CBF, we employ a non-invasive optical technique known as diffuse correlation spectroscopy (DCS). DCS offers many strategic advantages over more traditional modalities used to assess CBF (e.g., computed tomography, perfusion magnetic resonance imaging, and transcranial Doppler ultrasound), including portability, low cost, high temporal resolution, direct measure of microvascular blood flow, and ease of use in both adults and children in a wide array of clinical indications and environments. Indeed, several studies^42^^,^^49^^–^^57^ have explored the use of DCS to quantify the CBF response to a vasoactive stimulus (ACZ, HC). In this study, we use DCS to quantify CVR, and we hypothesize that CVR measured during HC will agree strongly with CVR assessed with more tolerable and more practical BH and RS paradigms.

## 2 Methods

Healthy adult subjects without a prior history of cardiovascular, neurological, respiratory, or hematological disorders were recruited at Emory University. Additional exclusion criteria included a history of major head injury within the past 2 years or a history of significant acute illness within one month of the study. Subjects were instructed to abstain from stimulants (e.g., caffeine) or depressants (e.g., alcohol) for at least 12 h prior to the study. Written informed consent was obtained for all participants. All protocols were approved by the Emory University Institutional Review Board.

Twenty-seven healthy adults (13 male) ranging in age from 22 to 37 years were enrolled in this study. A subset of four subjects was measured on multiple occasions, yielding 34 total measurement sessions. Of these sessions, one dataset was discarded due to substantial drift in optical signal attributed to poor sensor contact, and two datasets were discarded due to an improper execution of the HC exam, yielding a total of 31 available datasets.

### 2.1 Experimental Protocol

**Fig. 1:**
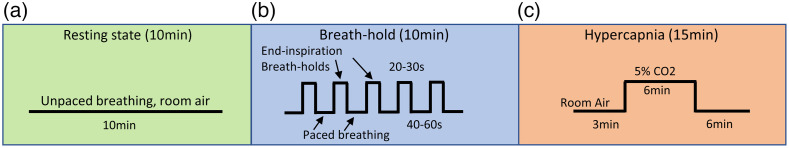
Experimental protocol: CVR was quantified using three consecutive experimental paradigms: (a) RS wherein the subject breathed naturally for 10 min, (b) BH, wherein the subject completed five end-inspiration BHs, and (c) HC, wherein the subject inhaled 5% ${\mathrm{CO}}_{2}$ for 6 min. Cerebral blood flow, ${\mathrm{EtCO}}_{2}$, peripheral oxygen saturation, and ABP were continuously monitored throughout the duration of the protocol.

The CVR was quantified via three consecutive experimental paradigms: RS, BH, and HC (Fig. 1). First, for RS, the subject was directed to breath naturally (i.e., unpaced) for 10 min while minimizing bodily movements. Next, for BH, the subject was asked to complete 5 BH challenges, each of which consisted of 40 to 60 s paced breathing followed by a 20 to 30 s end-inspiration BH. Paced breathing at the subject’s natural respiration rate (determined during RS) was facilitated by a custom graphical user interface written in MATLAB (Mathworks Inc., Natick, Massachusetts, United States). End-inspiration BHs, which have been demonstrated to yield similar CVR magnitudes when compared to end-expiration BHs,^38^ were selected to facilitate subject comfort during the exam. Subjects were instructed to breathe in approximately half capacity before initiation of the BH and to exhale entirely at the conclusion of the BH. Finally, for HC, the subject was instructed to breathe naturally for 3 min, followed by 6 min of 5% CO_2_/balance room air inhalation (Nexair, Memphis, Tennessee, United States) and 6 min of room air inhalation recovery. Unpaced breathing was used throughout the HC challenge.

Subjects sat upright for the duration of the study protocol. Continuous (20 Hz) DCS measurements of CBF were made by securing an optical sensor over the right or left forehead. Given that CVR is a global phenomenon,^58^ laterality of the sensor placement was determined based on the hemisphere that yielded the highest detected light intensities to maximize the DCS signal-to-noise ratio. Trends in peripheral oxygen saturation (SpO_2_, LifeSense II, Nonin Medical, Inc.) and arterial blood pressure (ABP, CNAP Monitor 500, NIPD1000D-1, Biopac Systems) were also continuously monitored (1 Hz for SpO_2_ and 125 Hz for ABP, Fig. S1A in the Supplementary Material). In postprocessing, beat-to-beat mean ABP (MAP) was determined from the ABP waveform using BP_annotate in MATLAB.^59^ Further, a gas mask was placed over the mouth and nose to facilitate continuous monitoring of expelled ${\mathrm{CO}}_{2}$ (4 Hz, LifeSense II, Nonin Medical Inc., Plymouth, Minnesota, United States) as well as to enable delivery of carbon dioxide during HC. For RS and BH paradigms, the mask was configured such that the subject breathed room air. For the HC challenge, the mask was attached to a reservoir of 5% CO_2_. One-way, non-rebreathing valves were used to maintain a constant inhaled CO_2_ concentration (Figure S1B and Table ST1 in the Supplementary Material). In postprocessing, a peak detection algorithm in MATLAB (findpeaks) was used to estimate end tidal CO_2_ (EtCO_2_) from the expelled CO_2_ waveform. The EtCO_2_ time series was then visually inspected to remove obvious outliers attributed to irregular breathing or incomplete exhalation.

### 2.2 Diffuse Optical Instrumentation

DCS measures of blood flow were acquired using a custom-built device consisting of a 852-nm-long-coherence near-infrared laser (iBeam Smart, TOPTICA Photonics, Farmington, New York, United States), two four-channel single photon counting modules (SPCM AQ4C-IO, Perkin-Elmer, Quebec, Canada), and an 8 channel counter/timer data acquisition board (PCIe6612, National Instruments, Austin, Texas, United States) that allowed for fast (20 Hz) quantification of the intensity autocorrection function, $g_{2}(t,\tau )$, at time, t, and delay time, τ.^60^

Frequency-domain near-infrared spectroscopy (fdNIRS) was also employed to assess baseline tissue optical properties (namely, the wavelength-dependent absorption and scattering coefficients, $\mu_{a}(\lambda )$ and $\mu_{s}^{\prime }(\lambda )$, respectively), as well as changes in $\mu_{a}(\lambda )$ over time.^53^ The fdNIRS system is a customized, commercially available device with 8 rapidly (20 Hz) multiplexed sources (690, 730, 750, 775, 785, 800, 825, and 830 nm) that are modulated at 110 MHz and four photomultiplier tube detectors with a gain modulation of 110 MHz + 5 kHz for heterodyne detection at 5 kHz (Imagent, ISS, Urbana Champaign, Illinois, United States). To assess baseline optical properties, measurements were made over the right and left frontal hemispheres with a rigid fdNIRS sensor containing one source and four detectors spaced at 2.0, 2.5, 3.0, and 3.5 cm.^53^ Resulting values were averaged to yield a global estimate of $\mu_{a}(\lambda )$ and ${\mu_{s}}^{\prime }(\lambda )$.

Continuous DCS + fdNIRS data were acquired with a flexible sensor containing one source fiber bundle and two detector bundles spaced 1.0 and 2.5 cm from the source ($\rho_{\text{short}}$ and $\rho_{\text{long}}$, respectively). The source bundle contained a 1000-μm multimode fiber for DCS (FT1000EMT, Thorlabs, Newton, New Jersey, United States) and three 400-μm multimode fibers for fdNIRS acquisition at 690, 800, and 830 nm (FT400EMT, Thorlabs). The $\rho_{\text{short}}$ detector bundle contained one single mode fiber for DCS (780HP, Thorlabs) and one multimode fiber for fdNIRS (FT600EMT, Thorlabs). The $\rho_{\text{long}}$ detector bundle contained seven single mode fibers for DCS (780HP, Thorlabs) and three multimode fibers for fdNIRS (FT600EMT, Thorlabs). Each source and detection fiber bundle was mechanically coupled to a 5-mm right-angle prism mirror (MRA05-E03, Thorlabs) using a custom 3D-printed prism/fiber holder. Prism/fiber holders were cast within a polyurethane mold (Vytaflex30, Smooth-On Inc., Macungie, Pennsylvania, United States colored black with PearlEx Carbon Black #640, Jacquard, Healdsburg, California, United States). The sensor was designed to be compliant with ANSI maximum permissible exposure standards of skin to laser radiation of $<4\;\mathrm{mW}/{\mathrm{mm}}^{2}$ at 852 nm.

### 2.3 DCS Data Analysis

Measured $g_{2}(\rho_{\text{long}},t,\tau )$ were first averaged across all seven detectors. Next, $g_{2}(t,\tau )$ at $\rho_{\text{short}}$ and the averaged $g_{2}(t,\tau )$ at $\rho_{\text{long}}$ were downsampled from 20 to 1 Hz to enhance the signal-to-noise ratio. These averaged and downsampled $g_{2}(t,\tau )$ curves were then fit for an index of blood flow using two separate models. First, $g_{2}(t,\tau )$ curves were fit to the semi-infinite homogenous solution to the correlation diffusion equation to extract an index of blood flow (BF_i_(*t*), ${\mathrm{cm}}^{2}/s$) at each source detector separation. For $\rho_{\text{long}}$, fits were restricted to $g_{2}>1.2$ to enhance the sensitivity to cortical blood flow.^49^ Note that, for our experimental configuration, $g_{2}$ ranged from 1 to ∼1.5. We opted to use 1.2 to improve the cortical sensitivity while still ensuring sufficient data points for fitting. For all $g_{2}$ fits, we assumed a fixed index of refraction of 1.4, and we incorporated fdNIRS-estimated, subject-specific $\mu_{a}(852\;\mathrm{nm})$, ${\mu_{s}}^{\prime }(852\;\mathrm{nm})$, and $\Delta \mu_{a}(852\;\mathrm{nm},t)$, as described elsewhere,^53^ to avoid the confounding influence of optical properties on BF_i_ estimation.^61^ When RS fdNIRS measurements were not available (N=3), group averaged optical properties were used. For each source detector separation, data were fit simultaneously for the coherence factor (β) and BF_i_ using fminsearchbnd in MATLAB with bounds for both β and BF_i_ set to [0.2, 0.55] and [1e-12, 1e-3] $\;{\mathrm{cm}}^{2}/s$, respectively.^51^

Next, to minimize extracerebral signal contributions, we also fit measured data from $\rho_{\text{long}}$ and $\rho_{\text{short}}$ simultaneously to a three-layer model^62^ (assuming scalp, skull, and brain) for scalp and brain blood flow (${\mathrm{SBF}}_{i}$ and ${\mathrm{CBF}}_{i}$, respectively) using a single cost function.^63^

$$\chi^{2}=\overset{N_{r}}{\underset{j=1}{\sum }}\overset{N_{\tau }}{\underset{k=1}{\sum }}[g_{2,\mathrm{meas}}(\rho_{j},\tau_{k})-g_{2,\text{fit}}(\rho_{j},\tau_{k},{\mathrm{SBF}}_{i},{\mathrm{CBF}}_{i}){]}^{2},$$ (1)

where $N_{r}$ is the number of detectors (2) and $N_{\tau }$ is the number of τ delay times. We minimize $\chi^{2}$ using fminsearchbnd in MATLAB with bounds for both CBF_i_ and SBF_i_ set to [1e-15, 1e-3] $\;{\mathrm{cm}}^{2}/s$. For these fits, we assumed negligible (0) flow in the skull and fixed values for layer optical properties and thicknesses according to the literature (Table 1).^64^ To improve the stability of fits, β for each separation was estimated by averaging the first five points of the autocorrelation curves.

**Table 1:** Assumed layer optical properties.

Layer	$\mu_{a}$ (${\mathrm{cm}}^{-1}$)	${\mu_{s}}^{\prime }$ (${\mathrm{cm}}^{-1}$)	L (cm)
Scalp	0.10	15	0.4
Skull	0.10	9	0.8
Brain	0.16	3	Inf

Assumed optical properties for absorption and scattering coefficients ($\mu_{a}$ and ${\mu_{s}}^{\prime }$) at 852 nm, as well as layer thicknesses (L) for scalp, skull, and brain.

For each experimental paradigm, the relative change in blood flow as a function of time (rCBF(t)) was calculated as $({\mathrm{BF}}_{i}(\rho_{\text{long}},t)-{\mathrm{BF}}_{i,0}(\rho_{\text{long}}))/{\mathrm{BF}}_{i,0}(\rho_{\text{long}})\times 100\%$ for the semi-infinite model and as $(C{\mathrm{BF}}_{i,\text{three}-\text{layer}}(t)-C{\mathrm{BF}}_{i,\text{three}-\text{layer,0}})/C{\mathrm{BF}}_{i,\text{three}-\text{layer,0}}\times 100\%$ for the 3-layer model. Here, the subscript 0 denotes the mean flow index during the baseline of the given paradigm. For RS, the baseline was chosen as the 2 min at the start of the RS period. For BH, the baseline was chosen as the 30 s prior to the first BH epoch. For HC, the baseline was chosen as the 3-min period of room air inhalation immediately prior to CO_2_ initiation. The change in EtCO_2_ as a function of time was estimated as $\Delta {\mathrm{EtCO}}_{2}(t)={\mathrm{EtCO}}_{2}(t)-{\mathrm{EtCO}}_{2,0}$ using the same baseline periods as rCBF. To account for the gas transit time from the mask to the capnogram and the physiological delay between alveolar diffusion of CO_2_ in the lungs and arterial CO_2_ reaching the cerebrovasculature, we time-aligned rCBF(t) and $\Delta {\mathrm{EtCO}}_{2}(t)$ via cross correlation. For this analysis, the EtCO_2_ timeseries was incrementally shifted at 0.01s intervals up to 10s. For each shift, $\Delta {\mathrm{EtCO}}_{2}(t)$ was interpolated to the rCBF(t) time axis, and Pearson’s correlation coefficient between rCBF(t) and $\Delta {\mathrm{EtCO}}_{2}(t)$ was estimated. Finally, $\Delta {\mathrm{EtCO}}_{2}(t)$ was shifted by the time lag that yielded the highest correlation coefficient between rCBF(t) and $\Delta {\mathrm{EtCO}}_{2}(t)$.

### 2.4 Estimation of CVR

We employed 2 numerical methods to estimate CVR (%/mmHg) for each experimental paradigm: (1) linear regression and (2) a general linear model (GLM) that regresses out systemic contributions to the DCS measured blood flow. The latter approach was included to minimize the known confounding extracerebral contributions to the DCS signal^50^^,^^65^ as well as the influence of heart rate and blood pressure variability.

#### 2.4.1 Linear regression

Linear regression is commonly used to assess CVR by modeling a simple linear relationship between rCBF(t) and $\Delta {\mathrm{EtCO}}_{2}(t)$:

$$\mathrm{rCBF}(t)=\beta_{0}+\beta_{1}*\Delta E{\mathrm{tCO}}_{2}(t)+Ɛ,$$ (2)

where $\beta_{1}$ reflects CVR (%/mmHg), $\beta_{0}$ is the intercept, and Ɛ is the fit residual. For each experimental paradigm, linear regression (regress, MATLAB) was applied between the cross-correlated, time-aligned rCBF(t) and $\Delta {\mathrm{EtCO}}_{2}(t)$ signals to obtain an estimate of CVR. To ensure that the model explains a significant amount of variability in the data, we discarded CVR estimates for which the model p-value for $\beta_{1}$ was >0.05. We further discarded any CVR value <0.1.

#### 2.4.2 General linear model

By the nature of the DCS measurement, detected light carries information about both cerebral and extracerebral (i.e., scalp and skull) hemodynamics. To minimize extracerebral contributions, we took a cue from best practices in functional near-infrared spectroscopy.^66^^–^^68^ We employed a GLM to regress out the relative changes in BF_i_ at $\rho_{\text{short}}$ (assumed to reflect scalp hemodynamics) from the relative changes in BF_i_ at $\rho_{\text{long}}$ (assumed to be sensitive to both superficial and brain layers), which is given as

$${\mathrm{rBF}}_{i}(\rho_{\text{long}},t)=\beta_{0}+\beta_{1}*\Delta E{\mathrm{tCO}}_{2}(t)+\beta_{2}*{\mathrm{rBF}}_{i}(\rho_{\text{short}},t)+Ɛ,$$ (3)

where $\beta_{1}$ reflects CVR (which we call ${\mathrm{CVR}}_{\mathrm{GLM}\text{-}\mathrm{SS}}$, %/mmHg) and $\beta_{2}$ represents the relative influence that the rBF_i_ at $\rho_{\text{short}}$ has on rBF_i_ measured at $\rho_{\text{long}}$.

We also explored the use of a GLM to regress out systemic contributions to the ${\mathrm{CBF}}_{i,3\text{-}\text{layer}}$ signal that are reflected in ${\mathrm{SBF}}_{i,3\text{-}\text{layer}}$. Here the GLM took the form of

$${\mathrm{rCBF}}_{i}(t)=\beta_{0}+\beta_{1}*\Delta EtCO_{2}(t)+\beta_{2}*rSBF_{i}(t)+Ɛ,$$ (4)

where $\beta_{1}$ reflects CVR (which we call ${\mathrm{CVR}}_{\mathrm{GLM},3\text{-}\text{layer}}$) and $\beta_{2}$ represents the relative influence that the scalp perfusion (${\mathrm{rSBF}}_{i}$) has on measured CBF index (${\mathrm{rCBF}}_{i}$).

CVR was estimated for each experimental paradigm using the cross-correlated, time-aligned signals by solving the system of linear equations utilizing the Moore-Penrose pseudoinverse (pinv MATLAB function). To ensure that the model explains a significant amount of variability in the data, we discarded CVR estimates when the model p-value for $\beta_{1}$ was >0.05. We further discarded any CVR value <0.1.

### 2.5 Statistical Analysis

Data are reported as median (interquartile range) unless stated otherwise. Paired Wilcoxon sign-rank tests were used to assess whether MAP, HR, and EtCO_2_ were statistically different from baseline for BH and HC paradigms. Wilcoxon rank-sum tests were used to assess differences in the change of these systemic parameters between HC and BH paradigms. To compare CVR across experimental paradigms, we performed Wilcoxon rank-sum tests between all combinations of paradigms for a given numerical method (e.g., linear-regression CVR compared between HC and BH), between all combinations of numerical methods for a given paradigm (e.g., HC CVR compared between linear regression and GLM methods), and between DCS analytical models for a given paradigm (e.g., linear regression HC CVR compared between semi-infinite and three-layer models). To quantify agreement of CVR across experimental paradigms, we calculate Pearson’s correlation coefficient (R) as well as Lin’s concordance correlation coefficient (CCC), which is a measure of how well a set of bivariate data compares to a “gold-standard” measurement (HC CVR, in our case).^69^ We define the following agreement thresholds for CCC: poor (0 to 0.5), moderate (0.5 to 0.7), strong (0.7 to 0.9), and excellent (>0.9) agreements. We further visualized agreement through Bland-Altman plots, and we quantified the mean bias (95% confidence interval).^70^ All statistical analyses were performed in MATLAB. Statistical significance was assessed at the 0.05 confidence level.

## 3 Results

As expected, EtCO_2_ significantly increased during both BH and HC paradigms (both p<0.05, Table 2). EtCO_2_ changes were more pronounced during HC (13.0 versus 7.5 mmHg in HC versus BH; p<0.001). During the RS paradigm, EtCO_2_ varied appreciably with a median range (i.e., max – min over the 10 min period) of 6.5 mmHg. MAP significantly increased by 8.0 (5.6, 11.6) mmHg during BH and 4.9 (1.9, 10.3) mmHg during HC (both p<0.001) (Table 2). Heart rate significantly increased by 11.5 (9.1, 19.2) bpm during BH (p<0.001); no changes were observed during HC.

**Table 2:** Systemic changes during each CVR paradigm.

	EtCO_2_ (mmHg)	MAP (mmHg)	HR (beats/minute)
	Delta from baseline		
**HC**	13.0 (7.80, 19.0)^+^*	4.9 (1.9, 10.3)^+^	2.1 (-3.1, 4.7)*
**BH**	7.5 (5.9, 9.2)^+^	8.0 (5.6, 11.6)^+^	11.5 (9.1, 19.2)^+^
	Range: median (IQR)		
**Resting state**	6.5 (5.3, 8.8)	1.9 (1.4, 2.3)	3.3 (2.9, 5.3)

Median (interquartile range) change in ${\mathrm{EtCO}}_{2}$, mean ABP (MAP) and heart rate (HR) for each experimental paradigm. For HC, this change reflects the steady state change from room-air inhalation, and for BH, this change reflects the peak increase during BH. For RS, the median (IQRs) of the range (max – min over the 10-min period) is reported. ^+^ denotes significant difference from baseline (p<0.05); * denotes significant difference between HC and BH (p<0.05).

**Fig. 2:**
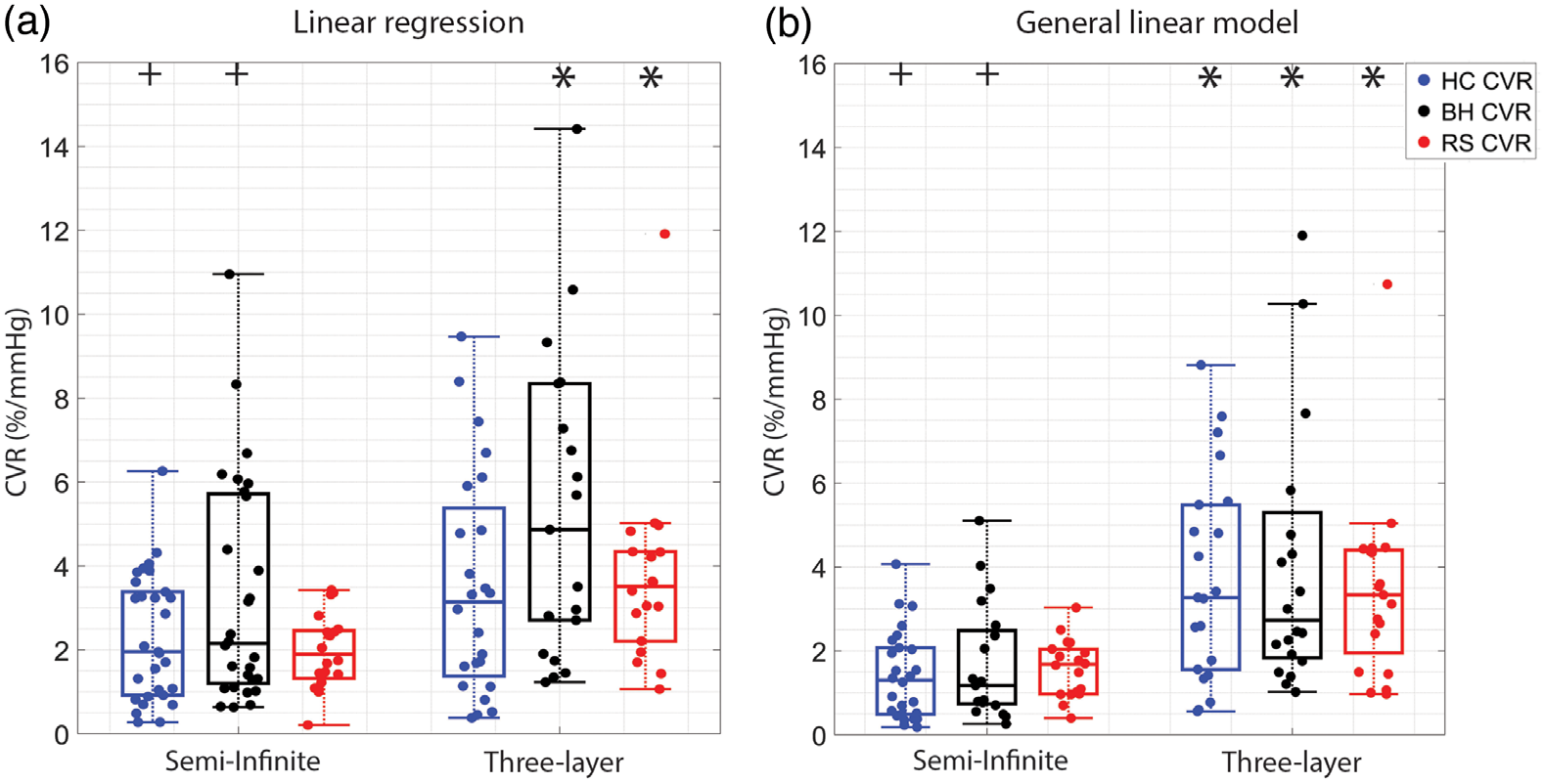
CVR across experimental paradigms: boxplots of CVR (%/mmHg) estimated with (a) linear regression and (b) a GLM using both semi-infinite and three-layer DCS analytical models during HC (blue), BH (black), and RS (red) experimental paradigms. For each boxplot, the central line denotes the median, and the bottom and top edges of the box indicate the 25’th and 75’th percentiles, respectively. The whiskers extend to the most extreme data points not considered outliers. Individual data are also included. * denotes significant difference between semi-infinite and three-layer for a given CVR paradigm and numerical method (p<0.05). ^+^ denotes significant difference between linear regression and the GLM estimate of CVR for a given experimental paradigm (p<0.05).

Of the 31 available datasets, a subset of CVR estimates for each experimental paradigm was discarded due to a failure to meet quality control criteria. The exact number of datasets included for each paradigm, along with median (IQR) CVR for each experimental paradigm and numerical method, is included in Table 3. The CVR was statistically indistinguishable when compared across experimental paradigms for a given numerical method (e.g., linear regression) or for a given DCS analytical model (e.g., semi-infinite, Table 3, Fig. 2). Within a given paradigm, the use of the three-layer model increased CVR compared with the semi-infinite model. Further, the use of a GLM with short separation regression depressed the median CVR and constrained the range compared with the linear regression approach.

**Table 3:** CVR across experimental paradigms.

	Linear regression		GLM	
	Semi-infinite	Three-layer	Semi-infinite	Three-layer
**HC**	1.95 (0.92, 3.38)^+^, 29	3.14 (1.48, 5.11), 24	1.30 (0.49, 2.06)^+^, 26	3.27 (1.55, 5.48)*, 21
**BH**	2.15 (1.24, 5.68)^+^, 28	4.86 (2.70, 8.34)*, 21	1.17 (0.73, 2.48)^+^, 19	2.73 (1.87, 5.03)*, 20
**RS**	1.89 (1.36, 2.44), 20	3.51 (2.37, 4.33)*, 18	1.67 (0.98, 2.02), 18	3.33 (1.95, 4.40)*, 19

CVR (%/mmHg) for HC, BH, and RS experimental paradigms estimated with linear regression and GLMs using the semi-infinite and three-layer models for DCS analysis. Data are reported as median (IQR), N datasets (out of 31) that passed quality control metrics. * denotes significant difference between semi-infinite and three-layer for a given CVR paradigm and numerical method (p<0.05). ^+^ denotes significant difference between linear regression and the GLM estimate of CVR for a given experimental paradigm (p<0.05).

**Fig. 3:**
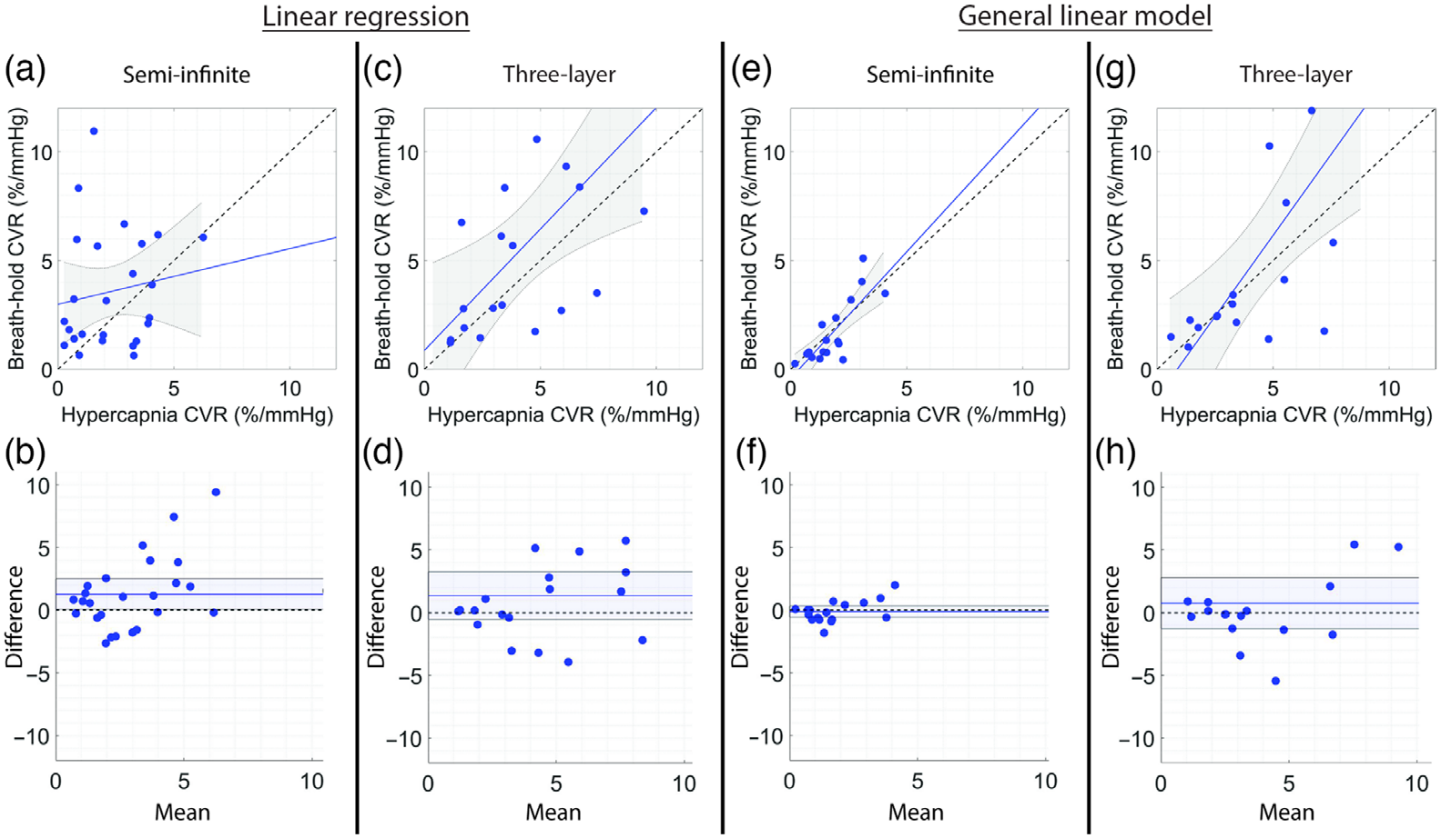
Relationship between HC and BH CVR. (a), (c), (e), and (g) Relationship between CVR estimated during HC versus BH and (b), (d), (f), and (h) corresponding Bland-Altman plots of the difference versus mean CVR estimated with linear regression (left columns) and GLMs (right columns) using the semi-infinite or three-layer model for DCS analysis. In panels (a), (c), (e), and (g), the dotted line represents the line of unity, and the solid line represents the best linear fit and 95% confidence interval (gray shaded region). In panels (b), (d), (f), and (h), the mean bias (solid line) and its 95% confidence interval (shaded region) are compared to the line of equality (dotted black line).

The relationship between CVR during HC versus BH is shown in Fig. 3 and summarized in Table 4. When using linear regression to estimate CVR, the correlation between HC and BH was only statistically significant for the three-layer model [R=0.58, p<0.01, Fig. 3(c)]; however, agreement was poor (CCC=0.45). No association between HC and BH was observed with linear regression for the semi-infinite model [Fig. 3(a)]. The strength of the HC versus BH CVR relationship greatly improved when using a GLM-SS to estimate CVR [Figs. 3(e) and 3(g)]. A strong correlation and agreement were observed using the GLM on the semi-infinite data [R=0.80, CCC=0.75, Fig. 3(e)], where the mean bias from the line of equivalence of −0.12%/mmHg was not significant [Fig. 3(f)]. Further, a modest correlation and moderate agreement were observed using the GLM on 3-layer data with scalp flow regression [R=0.69, CCC=0.52, Fig. 3(g)], with a non-significant mean bias of +0.76%/mmHg [Fig. 3(h)].

**Table 4:** Relationship between HC and BH CVR.

	HC versus BH			
	Linear regression		General linear model	
	Semi-infinite	Three-layer	Semi-infinite	Three-layer
N	25	18	17	16
R	0.15	0.58	0.80	0.69
p-value	0.48	0.0087	0.00011	0.0021
CCC	0.11 (−0.19, 0.39)	0.45 (0.14, 0.68)	0.75 (0.49, 0.88)	0.52 (0.24, 0.72)
Mean bias	1.28 (0.08, 2.49)	1.36 (−0.53, 3.25)	−0.12 (−0.57, 0.33)	0.76 (−1.28, 2.80)

Relationship between CVR estimated during hypercapnia versus BH using each numerical method and DCS analytical model corresponding to Fig. 3. N denotes the number of datasets that met quality inclusion criteria for both hypercapnia and BH, R denotes Pearson’s correlation coefficient and its corresponding p-value (p), CCC denotes Lin’s CCC with 95% confidence intervals, and mean bias (95% confidence interval).

**Fig. 4:**
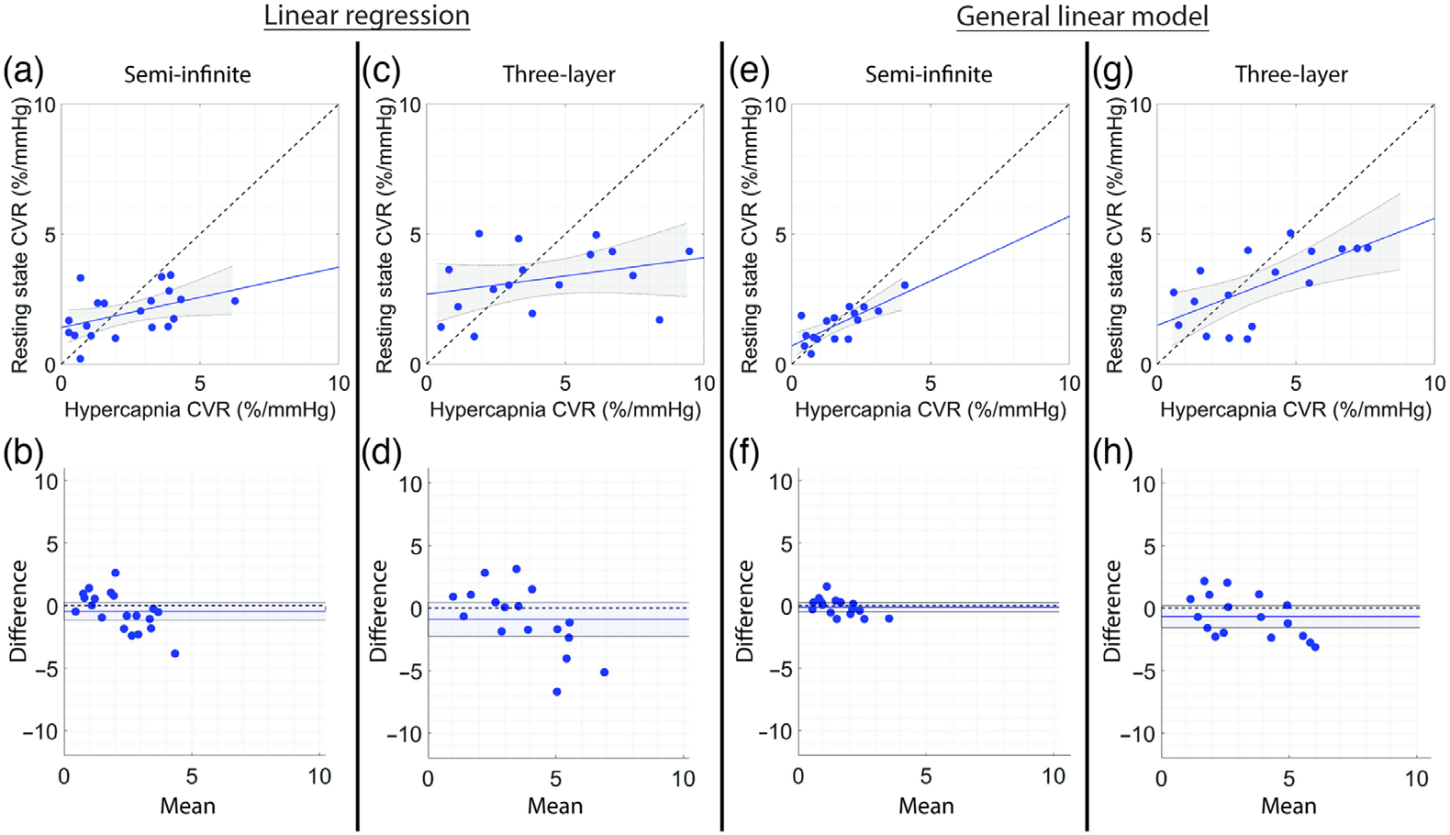
Relationship between HC and RS CVR. (a), (c), (e), and (g) Relationship between CVR estimated during HC versus RS and (b), (d), (f), and (h) corresponding Bland-Altman plots of the difference versus mean CVR estimated with linear regression (left columns) and GLMs (right columns) using the semi-infinite or three-layer models for DCS analysis. In panels (a), (c), (e), and (g), the dotted line represents the line of unity, and the solid line represents the best linear fit and 95% confidence interval (gray shaded region). In panels (b), (d), (f), and (h), the mean bias (solid line) and its 95% confidence interval (shaded region) are compared to the line of equality (dotted black line).

We next compared CVR agreement between HC and RS paradigms (Fig. 4, Table 5). When using linear regression to estimate CVR, the correlation between HC and RS was statistically significant, but the agreement was poor for the semi-infinite model [R=0.45, p=0.045, and CCC=0.35, Fig. 4(a)]; no association between RS and HC was observed for the three-layer model [Fig. 4(c)]. In a similar manner as the HC versus BH comparison, the strength of the HC versus RS CVR relationship greatly improved when using a GLM to estimate CVR [Figs. 4(e) and 4(g)]. A strong, statistically significant correlation and borderline strong agreement were observed using the GLM with short separation regression on the semi-infinite data [R=0.76, p<0.001, CCC=0.69, Fig. 4(e)] with a non-significant mean bias of −0.12%/mmHg [Fig. 4(f)]. A significant correlation and moderate agreement were observed using the GLM on three-layer data with scalp signal regression [R=0.65, CCC=0.55, Fig. 4(g)], with a non-significant mean bias of −0.68%/mmHg [Fig. 4(h)].

**Table 5:** Relationship between hypercapnia and RS CVR.

	Hypercapnia versus resting state			
	Linear regression		General linear model	
	Semi-infinite	Three-layer	Semi-infinite	Three-layer
N	20	17	16	17
R	0.45	0.31	0.76	0.65
p-value	0.045	0.23	0.00058	0.0048
CCC	0.35 (0.02, 0.61)	0.21 (−0.13, 0.51)	0.69 (0.40, 0.86)	0.55 (0.20, 0.77)
Mean bias	−0.46 (−1.17, 0.26)	−0.89 (−2.26, 0.47)	−0.12 (−0.50, 0.25)	−0.68 (−1.55, 0.19)

Relationship between CVR estimated during hypercapnia versus RS using each numerical method and DCS analytical model corresponding to Fig. 4. N denotes the number of datasets that met quality inclusion criteria, R denotes Pearson’s correlation coefficient and its corresponding p-value (p), CCC denotes Lin’s CCC with 95% confidence intervals, and mean bias (95% confidence interval).

## 4 Discussion

Herein we used DCS to quantify the agreement between CVR measured during HC compared with more tolerable and more practical BH and RS paradigms. Although these experimental paradigms are often used interchangeably in the literature as vehicles to assess CVR, we found surprisingly poor agreement between CVR assessed with each of these paradigms when using a simple linear regression to estimate CVR. This poor agreement was observed when using both the semi-infinite and three-layer models for DCS blood flow estimation. However, by employing a GLM to regress out extracerebral contributions, we found strong agreement between the “gold-standard” HC challenge and both BH and RS. This result suggests that the more CVR tolerable paradigms may serve as a substantially equivalent substitution for the HC challenge, thereby enabling bedside assessment of microvascular CVR with DCS in patient cohorts for whom HC is contraindicated or not feasible.

The use of a GLM with short separation regression (GLM-SS) is designed to remove extracerebral and systemic contributions to the long separation blood flow signal and, by extension, to enhance sensitivity to changes in brain-layer blood flow caused by dissolved CO_2_ in the bloodstream. Although commonly employed by the functional near-infrared spectroscopy (fNIRS) community to improve detection of evoked brain activity,^66^^,^^68^^,^^71^ this approach is novel in the assessment of CVR with DCS. Furthermore, this approach offers a unique advantage over other CVR-quantifying modalities, such as TCD, that lack easy access to information regarding systemic changes without the use of supplemental monitoring of ABP, heart rate, etc. Functional magnetic resonance imaging (MRI) commonly employs a global signal regression to account for systemic influences, among other contributors;^72^ however, when applied to the assessment of CVR, this approach yields only qualitative estimates that complicate inter-subject comparisons and is non-ideal for use in BH or HC.^73^ By employing short separation regression with DCS, we regress out extracerebral and systemic contributions to the signal in a manner that is comparatively more feasible than TCD and that can provide quantitative assessment of CVR across a wider range of experimental paradigms. We note that our implementation of the GLM-SS did not employ the use of a pre-whitening filter to minimize the influence of serially correlated (i.e., colored or heteroscedastic) noise.^74^ This filter was not used because (1) <25% of subjects exhibited oscillatory behavior in the temporal autocorrelation of error term, (2) prewhitening did not significantly change the estimation of CVR (e.g., for HC CVR with versus without pre-whitening, R=0.98, CCC=0.96 with an average difference of <0.12%/mmHg), and (3) prewhitening reduced the number of usable datasets (e.g., for HC, 23 datasets passed with pre-whitening vs. 26 without pre-whitening).

We have demonstrated a strong agreement among experimental paradigms using the GLM-SS approach; however, more work is needed to validate that CVR estimated with this approach reflects the true CVR. For reference, the literature commonly reports hypercapnic CVR in healthy adults to be ∼4 to 8%/mmHg.^35^^,^^55^^,^^58^ Our median (range) of 1.3 (0.5 to 4%/mmHg) is comparatively lower, possibly because the systemic influences that we regress out are known to be caused by factors that change CBF in tandem with the effects of dissolved CO_2_ (e.g., blood pressure and cardiac output). Alternatively, the blunted values may be due to the choice of source detector separations, which may regress out too much of the desired brain-layer BF_i_ signal due to a lack of optimal short separation or which may not have sufficient brain sensitivity due to the 2.5-cm-long separation. Finally, the blunted values could reflect that our assessments were made while the subjects were seated. Many studies, particularly with MRI, are performed with the subject in a supine posture, which is a known factor that increases the magnitude of CVR.^75^ Regardless, validation studies against other modalities (e.g., MRI) and clinical studies demonstrating the ability to detect abnormalities in CVR with this approach are warranted.

Although the strong agreement between HC and BH/RS with the GLM-SS approach is promising, we note that the overall rejection rate is high (23, 28, and 42% for HC, BH, and RS, respectively). We explored several explanations for this high rejection rate. For a subset of subjects (N=13/31), we observed that baseline BF_i_ ($\rho_{\text{long}}$) < BF_i_ ($\rho_{\text{short}}$), which may suggest a lack of brain sensitivity. For the RS paradigm, the ratio of BF_i_ ($\rho_{\text{long}}$)/BF_i_ ($\rho_{\text{short}}$) was significantly lower in rejected vs. passing datasets (0.81 versus 1.40; p<0.01); however, this trend was not observed for HC or BH paradigms. Further, in a subset of 6 subjects, we briefly applied pressure to the sensor to reduce scalp flow. Although the sample size was small, we observed that the relative change in BF_i_ at $\rho_{\text{long}}$ compared with $\rho_{\text{short}}$ during this pressure manipulation was higher but not significantly different in rejected data (2/6) across all paradigms (e.g., for RS, 0.88 versus 0.67). Taken together, these observations may suggest that the high rejection rate may be partially due to either regressing out too much brain signal with the 1 cm separation or not enough brain sensitivity with the 2.5-cm separation. The literature suggests that the latter is more likely as limited brain sensitivity is a well-known challenge in DCS measurements. Multiple ongoing, long-term efforts aim to address this brain sensitivity limitation;^76^ they include novel hardware developments (e.g., time-domain^77^^,^^78^ and interferometric DCS,^79^^,^^80^ along with moving to the second optical window^81^) and improved analytical approaches.^50^^,^^62^^,^^82^ As a mitigating near-term strategy, computational simulations are warranted to optimize source-detector separations, akin to what has been done with functional NIRS.^66^^,^^83^ For the RS paradigm, another explanation that we investigated for the high rejection rate was that the natural fluctuations in EtCO_2_ observed during unpaced breathing (typically a difference 1 to 3 mmHg on a breath-by-breath basis) may be too small to elicit reliable fluctuations in CBF, as has been previously suggested.^26^^,^^40^^,^^45^ However, median RS EtCO_2_ IQRs and beat-to-beat EtCO_2_ changes between accepted and rejected data were not statistically different (p>0.1), contradicting this previously offered explanation. Future work should be directed to better understand the factors that contribute to this high rejection rate to increase clinical viability of the CVR assessment with the GLM-SS approach.

Quantifying CVR with DCS is a particularly attractive opportunity for technology transfer as a diagnostic medical device for use in clinical settings given the unique economic, ergonomic, and clinical workflow integration benefits conferred by this optical neuromonitoring modality. Notable advantages include microvascular sensitivity in contrast to TCD, bedside portability in contrast to MRI, and ease of use in all patient cohorts in contrast to both TCD and MRI. Indeed, given these positive attributes, several studies^42^^,^^49^^–^^57^ have utilized DCS to quantify the CBF response to a vasoactive stimulus (e.g., ACZ, HC). Nevertheless, despite the advantages of DCS as a CVR-quantifying platform, drawbacks remain. The penetration depth of DCS is limited to the superficial cortex, and the spatial sensitivity is poor, unlike MRI, and as previously discussed, the measurement can be significantly confounded by the influence of extracerebral layers (i.e., skull, scalp, and cerebrospinal fluid). With regards to the latter limitation, we explored both the GLM-SS and the use of the three-layer model. With the three-layer model, we found that the magnitude of CVR significantly increased for most experimental paradigms and numerical approaches compared with the semi-infinite model, suggesting that the model did increase brain sensitivity. However, agreement among experimental paradigms was strongest with the semi-infinite model using the GLM-SS. The weaker agreement with the 3-layer model could be due to the sensitivity of this model to errors in the model’s assumed input parameters (Supplementary Material). For example, it has been shown to be sensitive to errors in both skull and scalp thickness, such that an underestimation of scalp thickness by ∼20% could induce ∼15% errors in the estimation of rCBF.^63^ To minimize these sources of error, future studies could benefit from concurrent anatomical scans (e.g., MRI or CT) to assess layer thickness and/or from the implementation of a realistic, multilayered Monte Carlo-based fitting process coupled with a pressure modulation paradigm to optimize model parameters.^50^

This study is not without limitations. First, the order of the experimental paradigms was not randomized by subject. Although we have no indication that order should influence agreement, the best practice to avoid unintended systematic error in experimental design would have been to randomize the order. Second, our estimation of CVR assumes a linear relationship between blood flow and end tidal CO_2_. This first-order approximation may break down outside the autoregulatory range of EtCO_2_ (∼35 to 45 mmHg), wherein the relationship between macrovascular blood flow velocity and EtCO_2_ has been shown to be logistic.^84^^,^^85^ Future work could benefit from quantifying the hemodynamic response function to step changes in EtCO_2_ to better account for possible non-linearity between CBF and EtCO_2_.^39^^,^^55^^,^^71^^,^^83^^,^^86^ Third, we did not rigorously control for environmental stimuli. Although the exact influence of the environment on our estimations of CVR is not well delineated, there is evidence to suggest that environmental stimuli can influence the brain’s response to changes in EtCO_2_. For example, Peng et al. showed that the time delay between the gray matter BOLD signal change and EtCO_2_ is larger with eyes-open versus eyes-closed states.^87^ The effect of environmental influences should be the subject of rigorous further investigation. Finally, there are numerous assumptions/approximations made when fitting for BF_i_ with the semi-infinite model and for CBF_i_ with the three-layer model (Supplementary Material). For example, with the semi-infinite model, we incorporate FDNIRS-estimated optical properties into the fitting procedure. The estimation of optical properties is predicated on several assumptions, e.g., ${\mu_{s}}^{\prime }$ does not change with time.^51^ For the three-layer model, we assumed that optical properties do not change with time, and we also used fixed, literature defined scalp and skull thicknesses for all subjects. Although the magnitude of the errors induced by each of these approximations in isolation may be small, the cumulative effect could be significant. Future work that targets this issue by minimizing the number of assumptions needed is merited.

## 5 Conclusions

This work provided the first systematic comparison of CVR across experimental paradigms using DCS. We demonstrated that using a GLM to regress out extracerebral and systemic contributions to the DCS-measured blood flow yielded a strong agreement between the “gold-standard” HC and the more tolerable and feasible paradigms of BH and RS. These findings suggest that BH and RS paradigms may offer feasible experimental alternatives to HC for practical clinical evaluation of CVR with DCS.
