# Supplementary material for: Agreement in cerebrovascular reactivity assessed with diffuse correlation spectroscopy across experimental paradigms improves with short separation regression
Source: Neurophotonics. 2023 Apr 7;10(2):025002. doi: 10.1117/1.NPh.10.2.025002 (PMC10079775; doi:10.1117/1.NPh.10.2.025002)
Supplement: Supplementary file 1 [file NPh_010_025002_SD001.pdf]

Agreement in Cerebrovascular Reactivity Assessed with Diffuse Correlation Spectroscopy Across Experimental Paradigms Improves with Short Separation Regression

Kyle R. Cowdrick[1], Tara Urner[1], Eashani Sathialingam[1], Zhou Fang[1], Ayesha Quadri[2,3], Katherine Turrentine[2], Seung Yup Lee[1,4], Erin M. Buckley[1,2,5, 1*]

[1]Wallace H. Coulter Department of Biomedical Engineering, Georgia Institute of Technology and Emory University 1760 Haygood Dr. NE, Atlanta, GA 30322, USA; [2]Department of Pediatrics, Children's Healthcare of Atlanta and Emory University School of Medicine, 1405 Clifton Rd. NE, Atlanta, GA 30322, USA; [3]Morehouse School of Medicine, 720 Westview Dr SW, Atlanta, GA 30310. [4]Department of Electrical and Computer Engineering, Kennesaw State University, 840 Polytechnic Ln, Marietta, GA 30060, USA. [5]Children's Research Scholar, Children's Healthcare of Atlanta, 2015 Uppergate Dr., Atlanta, GA 30322, USA.

Supplemental Material

Experimental Setup

Below we provide additional visualizations of the equipment utilized in this study for patient monitoring and delivery of 5% CO2 to the subject.

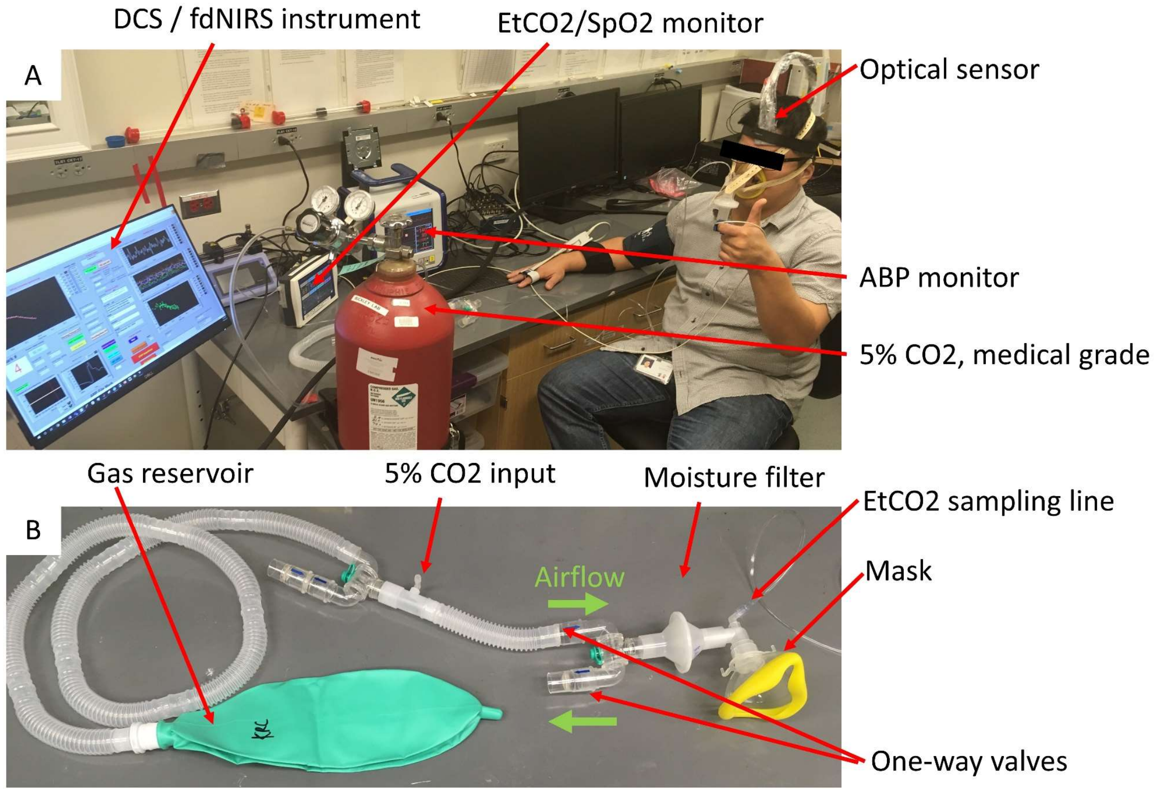

Figure S1. Experimental Setup and Respiratory Equipment
(A) Depiction of experimental setup with all monitoring instrumentation displayed. The respiratory mask is configured for the resting state and breath-hold paradigms. **(B)** Respiratory circuit configuration with relevant components annotated configured for hypercapnic gas inhalation.

[1] * *Address all correspondence to:* erin.buckley@emory.edu

Component	Manufacturer (Distributor)	Distributor Part Number
5% medical grade CO_2 (Balance N_2/O_2)	NexAir	SGV BG NICDOXC9-K
Standard anesthesia circuit tubing including:<br>Moisture Filter<br>EtCO_2 sampling line<br>Gas reservoir<br>T-Splitter	McKesson Medical Surgical	1016896
5% CO_2 input<br>Pressure Line Adaptor	Teleflex Medical (Medline)	HUD1642
One-way valves		HUD1665<br>HUD1664
Elbow adaptor with CO_2 sampling line		HUD1624
Standard Tubing Adaptors		HUD 1640
Masks	McKesson Medical Surgical	854706<br>854707<br>854708
Mask harnesses	Teleflex Medical (McKesson Medical Surgical)	336156<br>248120

Table ST1. Respiratory Circuit Components
Component, manufacturer, and part numbers are displayed for all pieces of the respiratory circuit.

Commentary on the sensitivity to model parameters

It is well-established that errors in optical properties and layer thickness can influence the accuracy of blood flow with DCS. We emphasize that these parameters do not affect the data fitting *per se*, i.e., the fits still converge when reasonable estimations are employed (**Figure S2**); however, the choice optical properties/layer thickness influences the value of CBFi. For example, in the 3-layer model, when $\mu_{s,brain}{}'$ is assumed to be 3 vs. 10 cm^{-1} the line of best fit changes minimally and fits still converge to reasonable estimations of CBFi, yet CBFi decreases by 66% (**Figure S2,** C-D).

For the semi-infinite model, errors in assumed μ_a and $\mu_s{}'$ can greatly confound estimations of absolute BFi, and errors in changes in $\mu_a(t)$ and $\mu_s{}'(t)$ from assumed baseline levels can influence estimation of relative changes in BFi (**Figure S2A,B**).[63] To mitigate these influences, we incorporate measured optical properties into our DCS fits. We note that we used the modified Beer-Lambert law (mBLL) for continuous monitoring of $\Delta\mu_a(t)$ because continuous estimation of $\mu_a(t)$ and $\mu_s{}'(t)$ with multi-distance FDNIRS was not possible due to sensor constraints. The mBLL assumes that changes in $\mu_a(t)$ are small and that $\mu_s{}'$ does not change significantly over the course of monitoring. The former assumption is supported by the values of $\Delta\,\mu_a(t)$ that we quantified. The latter assumption is supported by literature for the hypercapnia challenge[67]; this assumption also likely holds true for breath hold and resting state, as $\mu_s{}'$ is largely dominated by

organelles (mitochondria, cell nuclei, etc.), which should not change appreciably over the time scale of monitoring.[55]

For the three-layer model, errors in assumed brain μ_a and μ_s', as well as in skull and scalp thickness can greatly confound estimations of absolute CBFi.[65] Errors in assumed layer optical properties have negligible influence on relative changes in CBFi, assuming these properties do not change over the course of the measurement. It has yet to be rigorously quantified how errors in changes in layer optical properties from baseline levels influence the accuracy of relative CBFi estimations. Regardless, we do not have access to either layer optical properties or thicknesses for the 3-layer model, so our 3-layer results should be viewed with some caution.

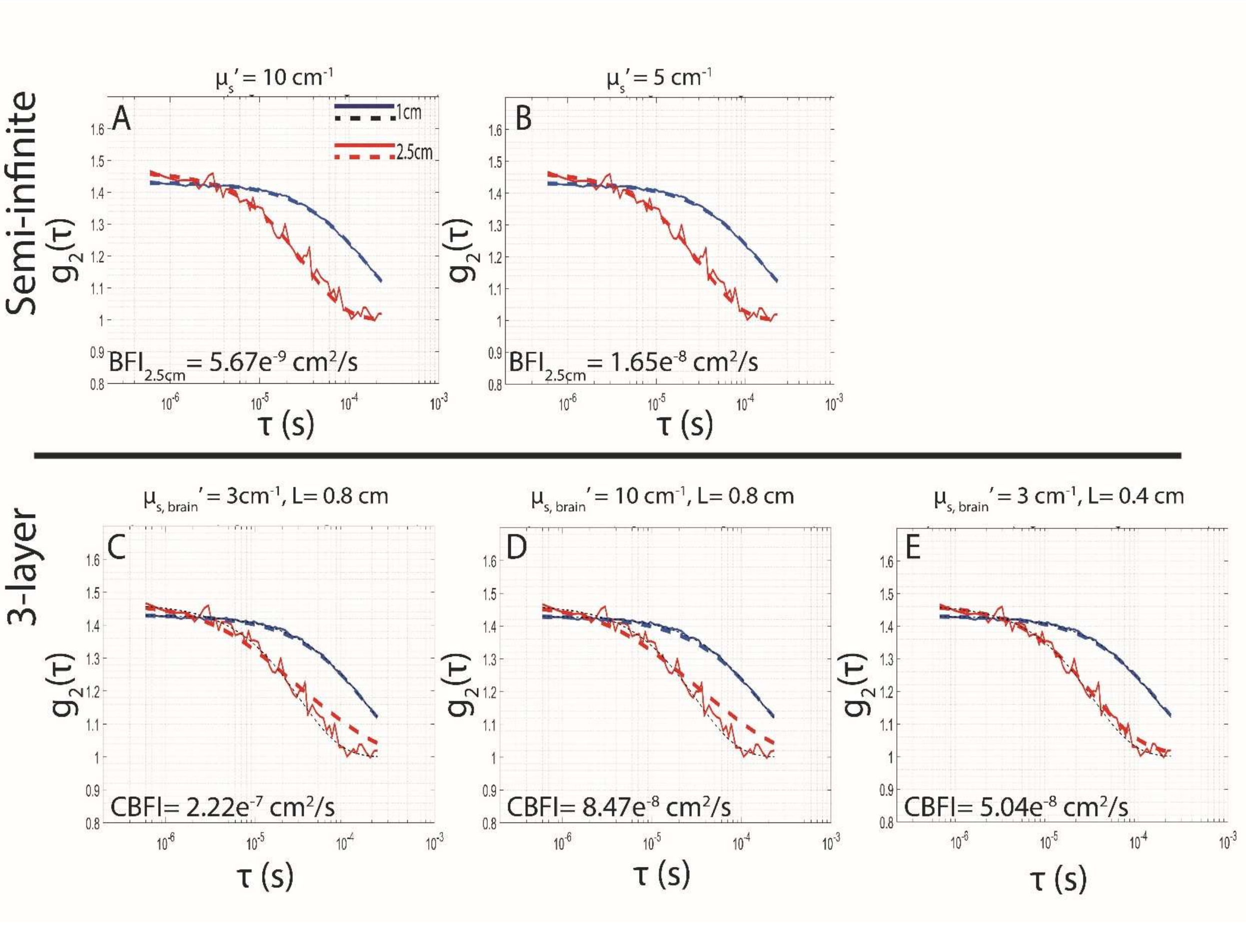

Figure S1. Influence of model parameters on data fitting. Representative measured g_2 data (solid lines) and corresponding best fit (dotted lines) for the short (blue) and long (red) separations for the semi-infinite model (A, B) and the 3-layer model (C-E). In A and B, all model parameters were held constant with the exception of μ_s', which was varied from 10 cm^{-1} (A) to 5 cm^{-1} (B). In C, D, and E, all model parameters were held constant with the exception of $\mu_{s,brain}'$ and skull-layer thickness (L). Further, a comparison to the semi-infinite fits is provided (thin black dotted line).
